# Ferocious Fighting between Male Grasshoppers

**DOI:** 10.1371/journal.pone.0049600

**Published:** 2012-11-14

**Authors:** Kate D. L. Umbers, Nikolai J. Tatarnic, Gregory I. Holwell, Marie E. Herberstein

**Affiliations:** 1 Research School of Biology, Australian National University, Canberra, Australia; 2 Evolution & Ecology Research Centre, University of New South Wales, Sydney, Australia; 3 Department of Biological Sciences, Macquarie University, North Ryde, Australia; 4 The School of Biological Sciences, The University of Auckland, Auckland, New Zealand; California State University Fullerton, United States of America

## Abstract

Contests among individuals over mating opportunities are common across diverse taxa, yet physical conflict is relatively rare. Due to the potentially fatal consequences of physical fighting, most animals employ mechanisms of conflict resolution involving signalling and ritualistic assessment. Here we provide the first evidence of ubiquitous escalated fighting in grasshoppers. The chameleon grasshopper (*Kosciuscola tristis*) is an Australian alpine specialist, in which males engage in highly aggressive combat over ovipositing females. We describe discrete agonistic behaviours including mandible flaring, mounting, grappling, kicking and biting, and their use depending on the individual’s role as challenger or defender. We show that male role predicts damage, with challengers being more heavily damaged than males defending females (defenders). Challengers also possess wider mandibles than defenders, but are similar in other metrics of body size. Our data suggest that fights escalate between males matched in body size and that mandibles are used as weapons in this species. This system represents an exciting opportunity for future research into the evolution of costly fighting behaviour in an otherwise placid group.

## Introduction

Although contests among individual animals over resources are common, they seldom escalate to physical combat. Theory predicts that less dangerous conflict resolution strategies should prevail because it is adaptive for potential combatants to avoid costly encounters whenever possible [1]–[3]. In species where competition is intense, rivals may exchange accurate information about each other’s competitive ability (resource holding potential) and should resolve contests before they escalate [4], [5]. While reliable indicators of fighting ability should resolve conflict between two poorly matched competitors with minimal cost to each party, the likelihood of costly, even lethal, consequences increases when competitors are closely matched in their ability to win and/or desire to obtain a resource [2], [3].

In many systems residency and body size asymmetries are important determinants of resource holding potential [6]–[8]. Where one competitor is larger than the other it is expected that the smaller competitor should retreat (assuming mutual-assessment) or be overpowered. For example, Wells (1988) showed that smaller jumping spiders (*Euophrys parvula*) lost 92% of contests [9]. In addition, the residency status of competitors can override the effect of size leading, for example, to the success of smaller residents [10]. This can occur: via the ‘bourgeois strategy’ [11], [12]; because residents have greater intrinsic resource holding potential than intruders; and/or because residents place greater value in their own territory than intruders [13]. Importantly, however, when the value of the resource is high (e.g. gravid females), contests are expected to escalate despite asymmetries [14].

Orthoptera (crickets, katydids and grasshoppers) are widely used in the study of conflict resolution. Crickets and katydids have provided classic examples of acoustic duelling and aggression and their influence on fitness [15]–[19]. For example, in field crickets (*Gryllus bimaculatus*) agonistic interactions escalate in the presence of females [14] and follow ritualistic displays through escalating interactions [20]. Also, in the fall field cricket (*Gryllus pensylvanicus*) males use their mandibles as weapons [21]. In rainforest katydids (*Gnathoclita sodalis*) agonistic interactions between males involve physical, acoustic and vibrational elements whereby the larger males win due to their size advantage and their vibratory regimes [22]. Grasshoppers (Orthoptera: Acridoidea), however, are known for their passive behaviour, leading orthopteran ethologist Dan Otte to conclusively state “grasshoppers do not fight” [23]. Since Otte’s groundbreaking work on grasshopper behaviour, there have indeed been no records of grasshoppers engaging in damaging physical fighting. To the best of our knowledge, reports of physical conflict in grasshoppers are limited to *Ligurotettix coquilletti*, in which one in five acoustic interactions result in grappling [24]. Instead of fighting, male grasshoppers usually use acoustic or visual cues to ritualistically determine and reinforce dominance hierarchies [23]. The chameleon grasshopper (*Kosciuscola tristis*), a robust, flightless acridid endemic to the Australian Alps, is atypical in that male contests escalate to potentially costly, damaging fights (see Supplementary Material). While females oviposit, males mount them but cannot mate because females’ abdomens are extended several centimetres into the soil. Over the course of oviposition further males attempt to usurp the mounted male. The mounted male defends his position while many other males challenge him.

In this study we observed and recorded fights over ovipositing females between male chameleon grasshoppers in the field. First we documented the types and pattern of behaviours that grasshoppers use when fighting. Secondly, we observed males fighting over females in the field and compared the behaviour and morphology of defenders and challengers testing the hypothesis that males’ roles in agonistic encounters predict their physical attributes. We predicted that defender males would be larger than challengers in one or more body size measures. Finally, we quantified the damage males exhibited as an approximate measure of the cost of fighting testing the hypothesis that their role in agonistic encounters predicts the amount of damage males have sustained. We predicted that defender males would have sustained greater amounts of damage through fending off challengers.

## Methods

### Field Observations of Fighting Behaviour

**Figure 1:**
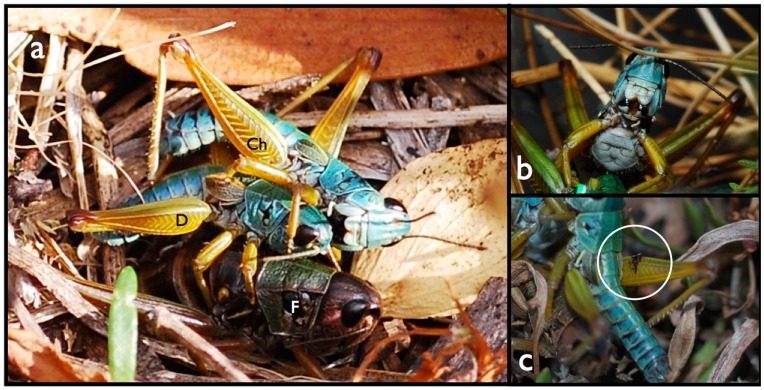
Common poses of grasshoppers during agonistic interactions. Panel (a) shows a defender (D) siting on the back of a female (F) while she is ovipositing and the challenger (Ch) attacks him and attempts to take his place. Panel (b) shows wing damage and tympanum exposed and panel (c) shows a grasshopper’s gape during mandible flare.

We observed *Kosciuscola tristis* grasshoppers at a site along the Dead Horse Gap walking track (36°30′14.0″S 148°16′36.7″E), Thredbo, NSW, Australia at 1961 m altitude. Male *K. tristis* commence fighting at approximately midday, and we conducted observations of 40 fights in a 20 m^2^ area between 11∶00 am and 5∶00 pm. When observers entered the area, focal ovipositing females were chosen as they were encountered, whether surrounded by males or not. Females and any surrounding males were observed for 15 minutes. Interactions between all males present were recorded. A male mounted on the back of a female was named ‘defender’, while ‘challengers’ were those attempting to usurp his position (Figure 1a).

During fights, males displayed several distinct behaviours: bite (mandibles engage with another grasshopper’s body), kick (hind legs in a sharp movement away from the body resulting in the propulsion of another), mandible flare (grasshopper arches back shakes head and opens mandibles), mount (grasshopper jumps from within 10 cm and lands along the anterio-posterior axis of the dorsum of another) and grapple (grasshoppers lock legs and roll around) (Figure 1, Table 1). We recorded the occurrence of these behaviours between all males present.

**Table 1:** The four most common male fighting behaviours are performed at different rates by defenders compared with challengers (n = 40 observations).

Type		Defender	Challenger	Statistics
Bite	mean±SD	1.53±2.89	0.80±1.76	Mann-Whitney: U = 724.5, z = 0.72, p = 0.47, n = 40, effect size r = 0.114
	range	0–12	1–9	
	median	0	0	
Kick	mean±SD	3.20±4.32	0.05±0.22	Mann-Whitney: U = 299.0, z = 4.82, p<0.01, n = 40, effect size r = 0.762
	range	0–20	0–1	
	median	2	0	
Mandible Flare	mean±SD	38.95±34.99	0	
	range	0–141	0	
	median	32.5	0	
Mount	mean±SD	0	1.70±2.40	
	range	0	0–12	
	median	0	1	

Once the observation time ended we collected the female, the defender and one haphazardly chosen challenger for morphometric analyses (pronotum length, weight, foreleg femur width and mandible width) and damage assessment. We made morphological measures with callipers and a proportion repeated to ensure accuracy [25]. Damage scores included injuries from past and present encounters, reflecting males’ overall fighting history. To ensure impartiality, the scorer did not know the status of the male (defender or challenger) when estimating damage. Injuries scored were: wing damaged/missing (1 per wing), hind leg damaged (1) and number of scars elsewhere (1 point per scar) (e.g. Figure 1c). We quantified damage to females using the same criteria. To compare the frequency of damage between defenders, challengers and females, we used a Friedman’s chi-square test. To assist in documenting and characterising stereotyped fighting behaviour, several fights were filmed (see Movie S1). Statistical tests were computed using SPSS Statistical Software (Version 19).

### Ethics Statement

No specific permits were required for the described field studies however, we did attain permits from New South Wales National Parks and Wildlife Service for collecting *Kosciuscola* grasshoppers in Kosciuszko National Park (License number S12256).

## Results

### Field Observations of Fighting Behaviour

By late March scores of female *K. tristis* were laying eggs at our observation site. Around midday, females with males riding on their backs (defenders) emerged from the surrounding foliage and descended to the bare earth of the study site. The females dug into the soil with their extended abdomens ovipositing an irregularly-shaped ootheca about 3 cm below the soil surface. Once females had begun laying, they could not move without disturbing oviposition. In many instances challengers aggregated around female/defender pairs and aggressively attacked the defender (Figure 1, see Movie S1).

**Figure 2:**
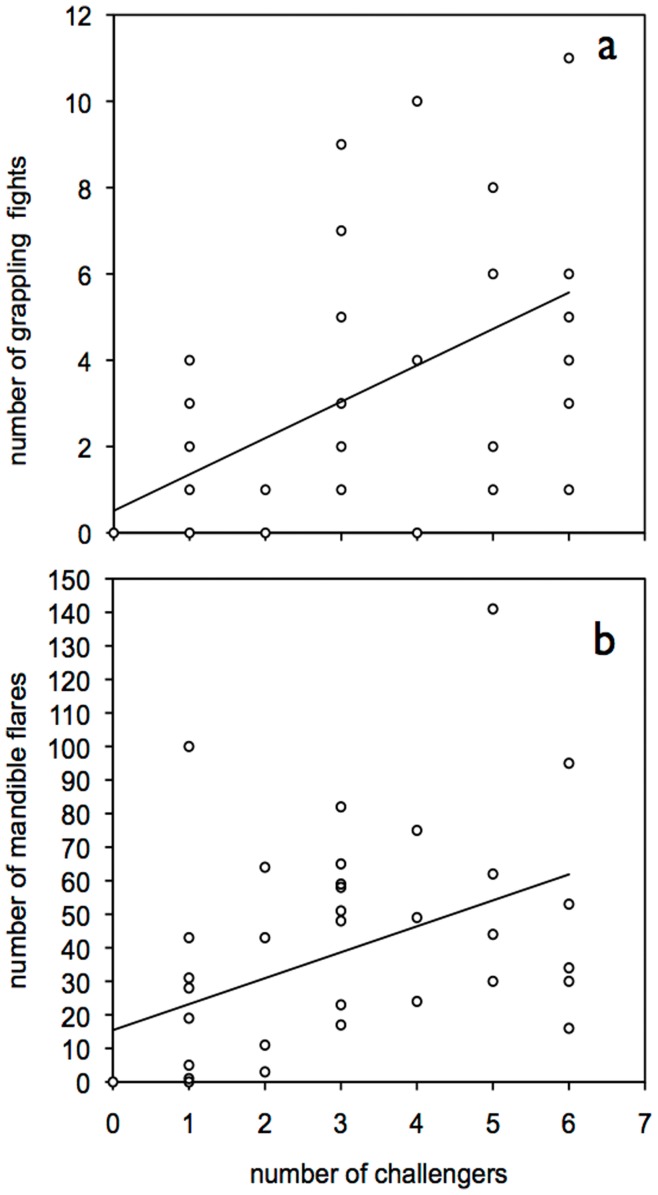
Trends in chameleon grasshopper agonistic interactions. Panel (a) shows that the number of grappling bouts between males increases with the number of males present (outlier removed) (with line of best fit). Panel (b) shows that defenders flare mandibles more often as the number of challengers increases (with line of best fit).

We observed 40 females ovipositing, each of which was mounted by a male. Six pairs had no challengers, while some had up to six (mean challengers per pair±SD = 2.83±2.06, n = 40, range = 0–6). The number of male-male grappling interactions observed during the 15 min observations increased with the number of males present (mean grapples±SD = 3.70±6.18, range = 0–37, Spearman’s ρ = 0.42, p = 0.007, n = 40). There was one obvious outlier (37), and after its exclusion the correlation became stronger (mean grapples±SD = 2.85±3.05, range = 0–11, Spearman’s ρ = 0.57, p<0.001, n = 39; Figure 2a).

During fights, we observed bites, kicks, mounts and mandible flares (the latter only executed by the defender) (see Movie S1). Challengers were frequently seen mounting other challengers and mounting the defender (Table 1). Defenders and challengers often exchanged bites (Figure 1a, Table 1), which in some cases caused immediate visible damage. Challengers sometimes bit other challengers, though this was rare (mean bites±SD = 0.08±0.35/15 min) compared to bites by challengers (mean bites±SD = 0.80±1.76/15 min) on defenders or defenders on challengers (mean bites±SD = 1.53±2.89/15 min; Table 1). To defend their position, defenders kicked challengers away with their hind legs, and challengers kicked significantly less often (Table 1). Defenders frequently reared back and flared their mandibles at challengers, but challengers never flared their mandibles at defenders (Figure 1b Table 1). During mandible flaring the defending male arched back and shook his head while expanding his white maxilla and labrum to expose his black mandibles and his mouth (see Movie S1). The number of mandible flares increased with the number of grappling bouts and with challengers present (Pearson’s r = 0.63, and r = 0.60, respectively, both n = 40, p<0.01; Figure 2b). Challengers often mounted defenders, who never responded in a like manner, as doing so would relinquish their position on top of the female (Table 1). For 35 of the 40 groups we were able to capture the female, the defender and one challenger. From this subset, we found that challengers had wider mandibles than defenders, but all other morphometric variables were not significantly different (Table 2).

**Table 2:** The differences and correlations between variables for defender and challenger males from field observations (average±SD).

	Defender	Challenger	Paired test	Correlation
Weight, n = 34	0.23±0.03 g	0.23±0.04 g	Mann-Whitney: U = 620, z = –0.51,p = 0.61	Spearman’s r = –0.16 p = 0.36
Pronotum Length, n = 35	3.62±0.18 mm	3.66±0.27 mm	Student’s t-test: t_34_ = 0.58,p = 0.57	Pearson’s r = 0.08, p = 0.67
Mandible Width, n = 35	2.21±0.09 mm	2.26±0.11 mm	Mann-Whitney: U = 784, z = –2.01,p = 0.04	Spearman’s r = –0.09, p = 0.96
Foreleg Femur Width, n = 35	1.08±0.04 mm	1.07±0.07 mm	Mann-Whitney: U = 520, z = 1.08,p = 0.28	Spearman’s r = 0.06, p = 0.73

### Are Fighters Damaged?

We quantified the number of melanised scars found on all participants collected from 35 of the interactions we observed in the field. The number of injuries was significantly different between defenders, challengers and females. The most damage was found on challengers, followed by defenders, then females (Friedman test: number of injuries: χ^2^_2_ = 7.87, p = 0.02; ranks: challenger = 2.33, primary = 1.89, females = 1.79; Wilcoxon’s post hoc tests: challengers v defenders: z = −2.26, p = 0.02, challengers v females: x = −2.76, p = 0.006, defenders v females: z = −1.04, p = 0.30).

## Discussion

Our study presents the first report of ubiquitous escalated physical fighting in a grasshopper species, with male chameleon grasshoppers fighting aggressively over ovipositing females. This is remarkable because physical fighting has not previously been reported in grasshoppers. Indeed in grasshoppers almost all species use ritualistic acoustic and/or visual signalling to resolve conflict [23]. The only other grasshopper known to exhibit any aggressive physical interactions is the tarbush grasshopper (*Ligurotettix planum*) [24]. In most cases male tarbush grasshoppers use acoustic signalling to resolve conflict. In around only 20% of contests for territory, conflict escalates to grappling between males [24]. Our observations of *Kosciuscola tristis* in the field however, show that aggressive interactions between males form a pervasive component of this species’ reproductive behaviour. Moreover, chameleon grasshopper aggressive encounters entail biting, kicking, mandible flaring and intense grappling and males readily escalate fights even under artificial experimental conditions but only in the presence of females (Umbers, unpublished data). We found no difference in measured aspects of body size between defenders and challengers (except with regard to mandible width) which may suggest that closely matched males enter into fights, however this assertion should be tested directly and in light of body size measures of non-challenging males.

### Mandibles as Weapons

Mandible flaring was the most striking and commonly observed behaviour in this study. The rate at which males used mandible flaring increased with the number of challengers faced, suggesting a role in aggressive signalling. The use of mandibles as weapons is relatively common in animal conflict [16], [21], [26]. For example, male tree weta (*Hemideina crassidens*) with larger mandibles can win fights against other males and gain larger tree cavities that contain more females [27]. In weta, as in chameleon grasshoppers, males display their mandibles in intraspecific aggressive and defensive behaviour [27]. Exactly what information mandible flaring conveys to a receiver is not clear, but it could reflect a male’s bite force and signal how much damage a challenger could sustain if he attacks [21], [28].

In our field observations, challenger males (who had larger mandibles) had sustained more physical damage compared with defenders. This may indicate that males with larger mandibles are injured more often, perhaps because they more readily participate in fights. This pattern is also found in fig wasps, where males with large mandibles sustain greater amounts of damage than males with smaller mandibles [29]. Given the frequency of mandible flaring and biting by male chameleon grasshoppers, we suggest that while mandibles are primarily used for grazing, they serve secondarily as weapons. Future studies should measure mandible shape, gape colour and width and bite force to see if these are predictors of contest outcome in this species.

Male chameleon grasshoppers fight over the top of ovipositing females. When we observe females ovipositing in nature, most of them have a male mounted on their dorsum. It is currently unclear whether females begin oviposition with a mounted male or whether males seek out ovipositing females and mount them. Regardless, males atop females, ‘defenders’, naturally fulfil the role of ‘residents’ and the ‘challengers’, ‘intruders’, as per the conflict literature [6], [8], [30]. This asymmetry causes defenders to match the aggression of challengers to if the defenders are to maintain their position on the female. While the current study does not attempt to decipher a residency effect in terms of the determinants of chameleon grasshopper contests, the defender’s residency advantage may explain why they maintained their position atop the female despite having smaller mandibles. It is clear that in future studies of this species, agonistic interactions must be viewed in light of residency asymmetry. For example, we expect that defenders (or residents) have a greater chance of winning against challengers (intruders) because: (a) from atop the female defenders have a mechanical advantage over challengers, (b) defenders as residents may have intrinsic qualities that lead to them being atop the female (holding the territory), and (c) defenders may have invested in the ‘territory’ i.e. the female, and thus have a greater knowledge of the resource (e.g. via previous mating events).

We speculate that the chameleon grasshopper’s great density in the field coupled with its short reproductive season enforced by the alpine environment may be drivers of the evolution of intense fighting in this species [31]. Given how unusual fighting behaviours are among grasshoppers, we feel that further research on the chameleon grasshopper’s conflict resolution (e.g. fitness costs and benefits) is warranted. Since combat is not thought to occur in other members of this genus, the *Kosciuscola* grasshoppers may represent an informative system with which to test hypotheses about the evolution of fighting behaviour in general. Furthermore, females might suffer reduced fitness as a result of males fighting on top of them (see Movie S1), either from direct injuries or a reduced ability to oviposit and forage. Male mating adaptations often reduce female or population fitness [32], and future studies could quantify this cost.

## Supporting Information

Movie S1 The first part of this recording shows a common bout of fighitng between male *Kosciuscola tristis*. Three males are primarily involved in the fight with four surrounding. During the fighitng the female’s oviposition is interrupted. The second recording shows a defending male and a challenger with the defending male using mandible display. The challenger attacks the defender and sucessfully usurps his position.
(MOV)
